# The impact of cognitive impairment of individuals with Parkinson’s disease on their caregivers’ mental health: A systematic review protocol

**DOI:** 10.1371/journal.pone.0271480

**Published:** 2022-07-19

**Authors:** Paulina Beata Golińska, Łucja Bieleninik, Michał Harciarek, Mariola Bidzan

**Affiliations:** 1 Department of Neuropsychology, Faculty of Social Sciences, Institute of Psychology, University of Gdansk, Gdansk, Poland; 2 Department of Clinical and Health Psychology, Faculty of Social Sciences, Institute of Psychology, University of Gdansk, Gdansk, Poland; 3 GAMUT-The Grieg Academy Music Therapy Research Centre, NORCE Norwegian Research Centre, Bergen, Norway; Clinca Geriatrica, ITALY

## Abstract

**Introduction:**

Parkinson’s disease is a motor disease, the second most common neurodegenerative disorder with cardinal symptoms including bradykinesia, rigidity, and rest tremor accompanied by cognitive difficulties. The caregivers play a crucial role for individuals with Parkinson’s disease; however, many of them may suffer from high caregiver burden and mental health deterioration. This protocol of a systematic review presents a methodology of the review about the impact of cognitive impairment of individuals with Parkinson’s disease on their caregivers’ mental health.

**Material and methods:**

Research will be identified by combining electronic databases searching and hand searching. The following databases will be included: Medline, PsycInfo, Web of Sciences, Cochrane, CINAHL, Embase and Scopus. The inclusion and exclusion criteria followed to PECOS model. The population of informal caregivers is defined as family members providing care on a patient with Parkinson’s disease. Exposure is linked with the evaluation of a cognitive functioning and outcome is defined as mental health among caregivers of individuals with Parkinson’s disease. We will include two types of studies: observational and intervention. Both, screening and eligibility will be done by two independent reviewers. Study quality will be assessed by two authors independently. Data will be extracted by two reviewers independently and will follow a pre-pilot extraction form. Any discrepancies will be resolved by discussion or/and consultation with another reviewer. The synthesis without meta-analysis (SWiM) guidelines will be used to report on included studies data. The metanalysis with usage the statistical software R version 4.1.2 (2021-11-01) “Bird Hippie” and R metaphor package 3.0–2 of will be conducted if possible.

**Discussion:**

The goal of this systematic review is to present the association between caregivers’ mental health problems and their proteges’ cognitive impairment. It will enable to identify the gaps in literature and its methodology giving the suggestions for further research.

**Protocol registration:**

Protocol registration number in PROSPERO: CRD42022296670

## Introduction

Neurodegenerative disorders are the significant source of disability requiring specialist care, like in a case of Parkinson’s disease being the second most common disorder [1]. In 2016, approximately 6,1 million individuals were diagnosed by Parkinson’s disease worldwide and the prognosis is growing [2]. Parkinson disease affects about 2% of men population and 1–3% women in USA, with a high variability of estimates worldwide and with a rising trend [3]. Prevalence of Parkinson’s disease cases increases more rapidly than other neurodegenerative disorders [4, 5].

For a long period of time, Parkinson’s disease has been considered as a typical motor disease [6]. The cardinal motor symptoms of Parkinson’s disease include bradykinesia, rigidity, rest tremor, and postural disability [7]. Currently, the disease picture is more complex and includes additionally nonmotor symptoms, such as: autonomic dysfunction, psychiatric disturbances, and cognitive decline [2]. Also, it has been demonstrated that in Parkinson’s disease the presence of both motor and nonmotor symptoms is related to dopamine deficiency in substantia nigra, which also impairs the functioning of the fronto-striatal networks [8, 9]. Variety of potential manifestations of nonmotor symptoms forms different disease profiles in patients [10]. Moreover, cognitive difficulties may appear only in some individuals, typically at more advanced disease stage. The severity of cognitive difficulties may change with the disease progression—from mild cognitive impairment, mild dementia to advanced dementia [11]. The profile of cognitive impairment in Parkinson’s disease is specific and differs from cognitive profile observed in other neurodegenerative disorders. For instance, Alzheimer’s disease is characterized by a relatively more global decline encompassing episodic memory impairment, visuospatial problems, language disturbances and dysexecutive syndrome [12, 13]. Previous studies conducted among caregivers of individuals with Alzheimer disease indicated that cognitive decline is strongly related to worsening or lack of dependency in activities of daily living and considered as crucial manifestation of neurodegenerative disorders contributing to caregiver’s burden and mental health impairment [14, 15].

In the light of constantly aging population, neurodegenerative disorders are important social problem, especially with regard to resources needed for providing the financial, organizational, and psychological care [16]. The costs of dementia are enormous worldwide and the economic burden increases with the growing prevalence of dementia. Only in United States, in 2015 the costs of care of dementia have reached $818 billion, giving an increase of 35% in comparison to 2010. The same study suggests that costs of informal and social care constitute similar proportions of dementia care total costs [17], what points to the role of family members in the process of caregiving. Importantly, for Parkinson’s disease alone, in 2017 the costs for a total of only diagnosed individuals with this disease in the U.S. reached $51.9 billion [18].

It has been proven that caregiving may be an overwhelming challenge for people providing informal care (not being the healthcare professionals or not having the medical background) [19]. The role of caregiver becomes more challenging and important in case when their care recipients with Parkinson’s disease experience cognitive impairment or dementia [20]. In many caretaking relatives, the high responsibility and expectations may cause feeling of burden and mental health adverse outcomes such as the presence of depression, high anxiety level, and strain [21, 22]. Therefore, family informal caregivers of people diagnosed with neurodegenerative disorders are still at the scientists’ interest center, whose aim is to identify the most important factors contributing to higher burden and mental health problems. This, in turn, would allow for implementing the most accurate therapeutical and psychoeducational programs as a way of help and support.

Considering the growing interest in caregivers’ mental health, high prevalence of Parkinson’s disease worldwide as well as the disease associated cognitive decline and dementia, it is relevant and interesting to evaluate whether there is an impact of cognitive difficulties of individuals with Parkinson’s disease on caregivers’ mental health. According to the authors best knowledge, there is no systematic review nor meta-analysis on impact of cognitive impairment of individuals with Parkinson’s disease on their caregivers’ mental health. To date, only one critical review was published [23]; however, this paper is limited in its scope on caregivers’ burden phenomenon in Parkinson’s disease. Therefore, there is still a need for a rigorous systematic review in which the impact of cognitive impairment in individuals with Parkinson’s disease on their caregivers’ mental health is analyzed. Thus, the aim of this paper is to present the protocol of aforementioned systematic review.

## Material and methods

The protocol has been created according to the Preferred reporting items for systematic review and meta-analysis protocols (PRISMA-P, S1 File) [24] and was registered with the International Prospective Register of Systematic Reviews (PROSPERO) database (registration number: CRD42022296670). The following main research question has been posted: How does the cognitive impairment of individuals with Parkinson’s disease impact on their caregivers’ mental health?

In line with the aforementioned research question, the objective of this systematic review is to assess the impact of cognitive impairment in individuals with Parkinson’s disease on caregivers’ mental health.

### Eligibility criteria

Studies will be selected according to the eligibility criteria outlined below.

Population: We will include studies of informal caregivers defined as family members providing care on patient with Parkinson’s disease. The individuals with Parkinson’s disease must be diagnosed with idiopathic Parkinson’s disease, confirmed by neurologists according to DSM-5 [25], or similarly as based on earlier versions of the DSM or the International Classification of Diseases–ICD [26]. We will exclude studies of informal caregivers of atypical Parkinson’s diseases due to different cognitive profiles in this population.

Intervention/Exposure: The review will include studies regardless of the type of intervention. By the term “cognitive functioning” we understand all cognitive processes such as attention, memory, visuo-spatial skills, gnosis, praxis, language not excluding executive functions. We will include studies with two approaches to cognitive functioning measurement. Firstly, studies, in which the cognitive status of individuals with Parkinson’s disease was assessed objectively with neuropsychological tests (not based on declarative information of caregivers) and secondly, studies, in which caregivers were asked to declare about the care recipient with PD cognitive status. The methods, which could be potentially used in studies with objective neuropsychological assessment were inter alia: Montreal Cognitive Assessment (MoCA) [27], Addenbrooke Cognitive Examination III (ACE-III) [28], Rey-Osterrieth complex figure [29], Californian Verbal Learning Test, (CVLT) [30], Clock Drawing Test [31], Verbal Fluency test [32]. The studies, in which only Mini-Mental State Examination was used to assess cognitive status will be excluded, since this measure is not a recommended screening tool for Parkinson’s disease and the results might be misleading [33].

Comparator/Control: Does not apply in this systematic review.

Outcomes: In this study the outcome is defined as mental health among caregivers of individuals with Parkinson’s disease. We aim to include the following outcomes: caregiver depression, caregiver anxiety, caregiver stress, caregiver burden, caregiver distress, caregiver burnout, caregiver strain, and remaining related terms.

Study design: We will include prospective cohort studies and cross-sectional addressing caregivers’ mental health and prospective studies of intervention effects with a control group (both randomized and non-randomized controlled).

Location: We do not impose any restrictions on the area of the conducted research.

### Search strategy and information sources

Research will be identified by combining electronic databases searching and hand searching. The following electronic databases will be screen by one reviewer: Medline, PsycInfo, Web of Sciences, Cochrane, CINAHL, Embase and Scopus. The search will not be restricted to language (only if an English language translation of the abstract will be available), sample size or year of publication. In case of non-English publications, we will contact corresponding authors via correspondence e-mail to clarify the compliance with the inclusion criteria. Unpublished studies such as conference abstracts, book chapters, books, dissertation will be excluded. Reference lists of the included original articles along with existing systematic review papers will be checked to identify additional studies.

Literature search strategies will be developed using Medical Subject Headings or equivalent along with the free text word terms/words related to the Parkinson’s disease and potential consequences for caregivers’ mental health. These terms will be implemented using the Boolean operators “and/or” along with proximity operators (parentheses and quotations) for each database. Th search strategy was piloted for PubMed database (see S2 File) in order to find the most relevant keywords. The search will be supervised by a specialist librarian if needed.

### Study selection

One reviewer will search electronic databases and handsearch the reference list of the included review articles and original articles. All references from each electronic databases will be extracted to EndNote. At this stage, duplicate reports of the same study will be detected and removed. Screening will be done by two independent reviewers, based on titles and abstracts. Disagreement between them will be resolved by discussion or in consultation with a third reviewer if needed. In the second step, full-text articles will be obtained and assessed by two reviewers independently. Disagreement between reviewers will be resolved by discussion. If no agreement can be reached, third reviewer will support and mediate the decision of the study final inclusion or exclusion. When needed, we will contact study investigators to clarify the study eligibility. Both screening and eligibility will be done in a standardized manner following *a priori* eligibility criteria. Inclusion and exclusion criteria will be piloted on a sample of reports to ensure that the criteria can be applied consistently by two reviewers. At this stage, information about exclusion will be gathered and reported. Eligibility criteria for each study will be assessed in order of importance, so that the first ‘no’ response will be reported as the primary reason for exclusion of the study, and the remaining criteria will not be assessed. Agreement between two reviewers will be calculated by using K statistics [34]. At the final step of the selection process, the care will be undertaken to link together multiple reports of the same study. One reviewer will check the following items for comparing reports: author names; location and setting, specific details of the interventions (for interventional research), numbers of participants and baseline data; and date and duration of the study. We will contact a corresponding author if uncertainties remain. All studies that meet the eligibility criteria will be included in the systematic review. Selection process will be presented on the PRISMA flow chart [35].

### Data extraction

An electronic data extraction form will be created in advance and piloted on a sample of report to check the existence of coding instructions for the data collection form. Data will be retrieved by two authors extracting the data from the eligible papers independently. Disagreements between them will be resolved through discussion or in consultation with a third reviewer. In addition, we will contact a corresponding author via e-mail to obtain missing data or resolve concerns if necessary. In case of multiple reports of the same study, we will extract data from each report separately and combine information across multiple data collection forms afterwards. After study results are split according to clinical characteristics, we will retain subgroups assigned prospectively, e.g. to intervention vs. control, or divisions between clinical characteristics and cognitive outcome measurement, because they might contain important information on heterogeneity. We will extract baseline data regardless of number of time points.

#### Data items

The following information will be extracted from the studies:

1. Author last name and year of publication
2. Country
3. Study design (O–observational; I- interventional)
3a In case of observational: cohort studies vs. cross-sectional
3b In case of interventional: randomized vs. non-randomized controlled
3c Intervention type
4. Follow up: duration in months and attrition rate.
5a Sample size of individuals with Parkinson’s disease (N of participant analyzed)
5b Sample size of caregivers (N of participant analyzed)
6 Demographic characteristics of Parkinson’s disease including:
6a age (M, SD, range as reported)
6b sex (% of male)
6c Parkinson’s disease duration (in months)
7 Demographic characteristics of caregivers including:
7a age (M, SD, range as reported),
7b sex (% of male),
7c kinship with the individual with Parkinson’s disease (as reported) (% of spouses, wife, children, siblings)
8. Clinical characteristics
8a severity of the disease measured with UPDRS (M, SD, range as reported)
8b severity of the disease measured with Hoehn-Yahr scale (M, SD, range as reported)
8c Levodopa daily dose (M, SD, range as reported)
9 Cognitive outcome measurement

The examples of outcomes we expect to find in studies are as follows:

Montreal Cognitive Assessment (M, SD, range, min. and max es reported)
Addenbrooke Cognitive Examination III (M, SD, range, min. and max es reported)
Rey-Osterrieth complex figure (M, SD, range, min. and max es reported)
Californian Verbal Learning Test (M, SD, range, min. and max es reported)
Clock Drawing Test (M, SD, range, min. and max es reported)
Verbal Fluency test (M, SD, range, min. and max es reported)

Furthermore, we will also collect all data from other tools, since we can not make provision for all types of tests used in studies on this level.

### Outcomes and prioritization

In this systematic review, the outcome is defined as mental health among caregivers of individuals with Parkinson’s disease. Systematic review and meta-analysis (if possible) will be performed for the following main outcomes: caregiver’ depression, caregiver’ anxiety, caregiver’ stress. We will collect data on the following secondary outcomes: caregiver burden, caregiver distress, caregiver burnout, caregiver strain. We expect mainly the correlation coefficients between outcomes and cognitive functioning as the results given in the papers.

### Risk of bias and study quality evaluation

The risk of bias will be assessed independently by two reviewers. Any concerns or discrepancies will be resolved by consensus between authors or/and in consultation with reviewer specialized in methodology and statistics if needed. We will use two different measurement tools to assess quality of study in individual studies depending on the study design. In case of observational studies (cohort and cross sectional) we will use NIH quality assessment [26]. We will use all 14 criteria included in original Quality Assessment Tool for Observational Cohort and Cross-Sectional Studies. Quality will be rated as poor for 0–4 questions, fair for 5–10 questions and good for 11–14 questions. In case of interventional studies, we will follow the Revised Cochrane Collaboration Risk of Bias Tool (RoB 2) for randomized trials [36] and the Risk Of Bias In Non- randomized Studies—of Interventions (ROBINS-I) tool for non-randomized studies [37]. Risk of bias in individual studies will be presented in a tabular manner represents three categories: high, low, or unclear risk bias.

### Strategy for data synthesis

The synthesis without meta-analysis (SWiM) guidelines is proposed as the most suitable approach facilitating transparent reporting on included studies data [38]. Screening will result in studies meeting inclusion criteria for each of the outcome we included in the review. Data synthesis for each outcome will be performed separately. In case of constructs overlapping, these will be combined and discussed together.

We suspect there will be limited scope for meta-analysis, subgroup analyses and meta-regression will be performed in meta-analysis if possible.

Continuous data will be recorded as the mean value. With usage of R Software (R Studio), we will present primary and secondary outcomes using forest plots in meta-analysis.

Statistical heterogeneity will be tested using the statistic *I*^2^, where from 0% to 40% might not be important; from 30% to 60% may represent moderate heterogeneity; from 50% to 90% may represent substantial heterogeneity; and from 75% to 100% stands for considerable heterogeneity. Pooling of outcomes across studies will be conducted using random-effects models. The pooled data will be computed using DerSimonian-Laird method. The 95% confidence interval will be estimated for the dichotomous outcomes if needed. Each outcome will be combined and calculated using the statistical software R version 4.1.2 (2021-11-01) “Bird Hippie” and R metaphor package 3.0–2 [39]. Subgroup analyses will be used to explore possible sources of heterogeneity including the following categorical predictors/predictor levels

1. divisions between diagnostic groups participant; subtype of cognitive impairment (executive difficulties vs other cognitive problems such as memory or visuo-spatial impairment),
2. caregivers’ sex.

The sensitivity analysis will be performed based on the quality of publications to assess the robustness of the results.

## Discussion

This systematic review will allow for better understanding the processes behind the caregivers functioning and enable to implement appropriate psychological and educational interventions. Previous studies demonstrated the adverse outcomes of mental health (inter alia depression and stronger stress) in caregivers of individuals with dementia [40, 41]. However, there is lack of clear evidence of similar relationship in caregivers of people with Parkinson’s disease; this seems especially important since the cognitive profile of individuals with Parkinson’s disease differs significantly from that of Alzheimer’s disease. It is relevant to obtain strong evidence that “dysexecutive profile” typical for most patients with Parkinson’s disease may lead to caregivers’ mental health struggles, likely different than in a case of Alzheimer’s disease. It is important for neuropsychologists’ clinical practice to learn who to support caregivers and family system as well as to establish predictors contributing to higher caregivers’ burden. There is also a need for psychoeducational programs supporting caregivers knowledge about providing care of individuals with Parkisnon’s disease. Hence, this study will fill the gap in knowledge about the impact of cognitive difficulties of affected individuals on their caregivers’ mental health and facilitate health care providers, researchers, and policy makers the rational decision making. The proposed review may also point out the methodology weaknesses and limitations as well as delineate directions for future studies.

## Supporting information

S1 File PRISMA-P 2015 checklist. (DOC)

S2 File PUBMED search strategy. (DOCX)
