# Supplementary material for: The impact of cognitive impairment of individuals with Parkinson’s disease on their caregivers’ mental health: A systematic review protocol
Source: PLoS One. 2022 Jul 19;17(7):e0271480. doi: 10.1371/journal.pone.0271480 (PMC9295953; doi:10.1371/journal.pone.0271480)
Supplement: S2 File — (DOCX) [file pone.0271480.s002.docx]

**PUBMED SEARCHING STRATEGY**

**Concept 1: Disease**

Keywords: "Parkinson’s disease"[tiab] OR "Parkinson Disease"[Mesh]

MeSH: "Parkinson Disease"[Mesh]

**Concept 2: Caregivers**

**Keywords:** caregiver*[tiab] OR "Caregivers"[Mesh]

MeSH: "Caregivers"[Mesh]

**Concept 3: Health problems**

"Depression"[Mesh] OR "Level of Stress"[tiab] OR "Anxiety"[Mesh] OR "Caregiver Burden"[Mesh] OR "Caregiver’s Burden"[tiab] OR Distress[tiab] OR Burnout[tiab] OR Stress[tiab] OR Strain[tiab] OR "Burnout, Psychological"[Mesh]

*#1 AND #2 AND #3*
